# Investigating the Potential of Native Soil Bacteria for Diesel Biodegradation

**DOI:** 10.3390/microorganisms13030564

**Published:** 2025-03-02

**Authors:** Mihaela Marilena Stancu

**Affiliations:** Institute of Biology Bucharest of Romanian Academy, 296 Splaiul Independentei, P.O. Box 56-53, 060031 Bucharest, Romania; mihaela.stancu@ibiol.ro

**Keywords:** native bacteria, alkane hydroxylase, catabolic genes, diesel, biodegradation

## Abstract

In countries with a long petroleum extraction and processing history, such as Romania, extensive soil areas are often polluted with petroleum and its derivatives, posing significant environmental and human health risks. This study explores the diesel biodegradation potential of two native bacterial consortia isolated from hydrocarbon-polluted soils, focusing on their phenotypic and molecular characteristics, growth kinetics, alkane hydroxylase activity, hydrolase production, and biosurfactant synthesis capabilities. The bacterial consortia, CoP1 and CoP2, were successfully obtained using the standard successive enrichment culture method from two soil samples collected from a region affected by petroleum pollution. The CoP1 and CoP2 consortia demonstrated efficient diesel-degrading capabilities, achieving 50.81−84.32% degradation when cultured in a minimal medium containing 1–10% (*v*/*v*) diesel as the sole carbon and energy source. This biodegradation potential was corroborated by their significant alkane hydroxylase activity and the detection of multiple catabolic genes in their genomes. The CoP1 consortium contains at least four catabolic genes (*alkB*, *alkM*, *todM*, *ndoM*) as well as rhamnosyltransferase 1 genes (*rhlAB*), while the CoP2 consortium contains only two catabolic genes (*ndoM*, *C23DO*). The RND transporter gene (*HAE1*) was present in both consortia. Secondary metabolites, such as glycolipid-type biosurfactants, as well as extracellular hydrolases (protease, amylase, cellulase, and lipase), were produced by both consortia. The CoP1 and CoP2 consortia demonstrate exceptional efficiency in diesel degradation and biosurfactant production, making them well suited for the bioremediation of soils contaminated with petroleum and its derivatives.

## 1. Introduction

Due to the expansion and intensification of industrial activity, many countries face serious environmental pollution problems caused by toxic organic compounds. An important category of pollutants consists of petroleum and its derivatives from the petroleum industry, which often contaminate soils, leading to harmful environmental consequences. Petroleum is a complex mixture of hydrocarbons, including linear, branched, and cyclic alkanes, as well as mono- and poly-aromatic hydrocarbons. It also contains non-hydrocarbon compounds, such as asphaltenes, resins, waxes, and tar [1,2]. Generally, soil pollution with hydrocarbons is less extensive than spills into water, and the spread of contaminants is influenced by several factors such as the soil slope, soil adsorption capacity, and vegetation cover. The fate of hydrocarbons in soil is influenced by their physical and chemical properties (e.g., water solubility, mobility, affinity for organic carbon) and soil characteristics (e.g., particle size, porosity, organic matter content, permeability). Soil pollution causes significant changes in its biological processes, chemical composition, structure, and physical properties. As a result, hydrocarbon contamination has important ecological and health risks due to its toxic, mutagenic, and carcinogenic effects [1,2,3,4,5].

Although biodegradation typically takes longer than traditional physicochemical remediation methods, it has the advantage of completely degrading petroleum and its derivatives. Bacteria degradation is a key approach for removing these contaminants from soil, and both biotic and abiotic factors generally influence its efficiency. Biotic factors, such as the structure of the bacterial community and the presence of metabolically active bacteria, can often limit the biodegradation process. Abiotic factors, such as the hydrocarbon characteristics (physical and chemical nature of the contaminant, concentration, bioavailability) and physical parameters (soil characteristics, nutrient level, oxygen availability or other electron acceptors, temperature, pH, humidity, salinity) often limit the biodegradation process by inhibiting the growth of bacteria capable of degrading hydrocarbons [1,4,6]. Given the nature of contamination in soil and the importance of bioremediation strategies, understanding the fate of hydrocarbons, especially their interaction with native bacteria, is essential [1,7,8,9,10].

Hydrocarbon-degrading bacteria are ubiquitously distributed in soils worldwide [6,11]. However, bacterial communities in chronically contaminated environments, such as petroleum fields, exhibit distinct physiological adaptations, including enhanced tolerance and degradation capabilities for hydrocarbons compared to those in unpolluted soils. It is well established that bacteria from polluted soils often develop mechanisms to tolerate and degrade these toxic compounds, such as employing efflux pumps, altering cell membrane compositions, activating enzymatic degradation pathways, and acquiring new genes through horizontal transfer [6,8,11,12,13,14]. Bacteria use efflux pumps to eject hydrocarbons from cells, reducing intracellular accumulation. Some bacteria alter their cell membrane composition to prevent hydrocarbon entrance and maintain cell stability [12]. Most bacteria can only degrade simpler hydrocarbons (alkanes or aromatics), whereas the ability to break down complex hydrocarbon mixtures is limited to a few specialized species. The complete degradation of hydrocarbon mixtures typically relies on the cooperation of various high-performing bacteria with complementary substrate specificities. In polluted soils, bacterial communities often form, with each member specializing in the degradation of specific hydrocarbons. Bacteria from these sites generally produce higher levels of specialized enzymes, such as oxygenases (monooxygenases and dioxygenases), and dehydrogenases that break down hydrocarbons. These bacteria also develop specialized metabolic pathways to use hydrocarbons as their sole carbon and energy source, often involving multi-step processes in which intermediates undergo further enzymatic breakdown to enhance hydrocarbon metabolism [5,8,11,14,15]. Polluted soils serve as hotspots for horizontal gene transfer, enabling bacteria to exchange genetic material, including catabolic genes involved in hydrocarbon degradation, thereby enhancing the metabolic potential of native bacterial communities [6].

Chronically contaminated soils sometimes have high salinity or low oxygen, adding stress to native bacteria. Adapted bacteria develop supplementary survival mechanisms, such as salinity resistance or anaerobic degradation pathways [16,17]. In addition, bacteria inhabiting polluted soils synthesize biosurfactants that serve diverse physiological functions, such as enhancing motility and chemotaxis, facilitating the localization and degradation of hydrocarbons, altering cell membrane hydrophobicity, promoting biofilm formation, and mediating quorum sensing. Like synthetic counterparts, biosurfactant molecules have a hydrophilic head and a hydrophobic tail, reducing the surface and interfacial tension. Their ability to partition across interfaces, such as liquid–liquid, liquid–solid, and liquid–gas makes them effective as emulsifiers, foaming agents, and dispersants. Through processes like pseudo-solubilization and emulsification, biosurfactants increase the accessibility of hydrocarbons to bacteria, thereby enhancing their biodegradation [2,14,18,19,20,21].

Among petroleum products, diesel oil (commonly referred to as diesel or diesel fuel) is a widespread energy source in the transportation and industrial sectors worldwide. Diesel is a petroleum-derived product formed during the fractional distillation of crude oil. It consists of a mixture of alkanes, including small-, medium-, and long-chain *n*-alkanes ranging from C8 to C40, and volatile or non-volatile aromatic hydrocarbons [8,22]. In recent decades, the utilization of native bacterial consortia, which combine the metabolic capabilities of different species, has emerged as a promising strategy for the efficient biodegradation of complex hydrocarbon mixtures [5,6,8,9,13,15]. However, diesel is primarily composed of alkanes (over 90%), with lower levels of aromatic hydrocarbons (below 5%). Most early studies focused on the fate of aromatic hydrocarbon pollutants in soil [23]. Medium-length *n*-alkanes found in diesel are generally less biodegradable since they are non-volatile, non-polar, water-insoluble, and have low bioavailability [24]. This study explores the diesel biodegradation potential of two native bacterial consortia isolated from hydrocarbon-polluted soils, focusing on their phenotypic and molecular characteristics, growth kinetics, alkane hydroxylase activity, hydrolase production, and biosurfactant synthesis capabilities.

## 2. Materials and Methods

**Isolation and characterization of native bacterial consortia.**

Bacterial consortia used in this study were isolated using the standard successive enrichment culture method from two hydrocarbon-polluted soil samples collected in Poeni, Teleorman County, Romania. Soil samples (5% *v*/*v*) were used to initiate enrichment cultures in minimal salt medium (MSM) supplemented with 5% (*v*/*v*) petroleum as the sole carbon and energy source. The composition of the MSM [25] was (g/L): K_2_HPO_4_, 4.4; KH_2_PO_4_, 1.7; NH_4_Cl, 2.1; and 10.0 mL stock salt solution containing (g/L) MgSO_4_, 19.5; MnSO_4_, 5.0; FeSO_4_, 5.0; CaCl_2_, 0.3; and acid ascorbic, 1.0. The pH of the MSM was adjusted to 7.2 since the pH of the soil samples was between 7.0 and 7.2. Tubes were incubated aerobically at 30 °C in a rotary shaker set to 200 rpm for 21 days. Here, as elsewhere in this work, the assays were performed in duplicate. The soil enrichment cultures (5% *v*/*v*) were transferred to fresh MSM supplemented with 5% (*v*/*v*) petroleum and incubated under the same conditions for an additional 21 days. Bacteria quantification in the enrichment cultures was performed using the 96-well microplate most probable number (MPN) method [26] and the conventional plate count agar method [10]. The isolated consortia were preserved at −80 °C in 25% (*v*/*v*) glycerol.

Before **phenotypic** and **molecular characterization**, the bacterial consortia were inoculated into LB broth [27] and incubated aerobically at 30 °C in a rotary shaker set to 200 rpm for 1–3 days. Then, several standard **physiological** and **biochemical** assays were conducted to determine the optimum growth temperature, pigmentation, Gram characteristics, cell morphology, motility, oxygen relationship, rhodamine 6G accumulation, production of pyocyanin and pyoverdin/fluorescein pigments, tolerance to TTC, lactose fermentation ability, and enzymatic activities (protease, lipase, amylase, and cellulase) [28]. 

The **hydrocarbon tolerance** of the bacterial consortia was evaluated using the hydrocarbon overlay agar method. Bacterial cultures (20 μL) were spotted onto LB agar [27], air-dried, and overlaid with various hydrocarbons, such as diesel, gasoline, kerosene, *n*-alkanes (*n*-hexadecane, *n*-pentadecane, *n*-decane, *n*-heptane, and *n*-hexane), and aromatics (xylene isomers, ethylbenzene, and toluene). Hydrocarbons were removed from the surface of the Petri plates after 3 h. Control tests without hydrocarbons were conducted under similar conditions. The Petri plates were incubated at 30 °C for 1–2 days, and hydrocarbon tolerance was determined based on bacterial growth (colony formation) relative to the controls. Here, as elsewhere in this work, the Petri plates were observed under visible light (500 nm) and ultraviolet (UV) light (366 nm).

For **molecular** characterization, genomic DNA was extracted from the bacterial consortia using the PureLink Genomic DNA Kit, as recommended by the manufacturer (Invitrogen, Carlsbad, CA, USA). PCR amplification of the 16S ribosomal RNA (rRNA) gene, catabolic genes (*alkB*, *alkM*, *todM*, *xylM*, *ndoM*, and *C23DO*), RND transporter (*HAE1*), and rhamnosyltransferase 1 (*rhlAB*) genes was performed using genomic DNA (0.5 μg), specific primers (27f/1492r, [29]; alkB-f/alkB-r, [30]; alkM-f/alkM-r, todM-f/todM-r, xylM-f/xylM-r, ndoM-f/ndoM-r, [31]; 23CAT-f/23CAT-r, [32]; A24f2/A577r2, [33]; rhlA-f/rhlB-r, [34]), and GoTaq G2 Flexi DNA polymerase, as recommended by the manufacturer (Promega, Madison, WI, USA). PCR amplification of the target genes was performed using an Eppendorf Mastercycler Pro S thermocycler (Hamburg, Germany). The PCR program comprised an initial denaturation step (94 °C for 10 min), followed by 35 cycles of amplification (94 °C for 1 min, 50–62 °C for 30 s, 72 °C for 2 min), and a final extension step (72 °C for 10 min). The annealing temperature was 55 °C for the 27f/1492r primers; 60 °C for the alkB-f/alkB-r primers; 62 °C for the alkM-f/alkM-r, todM-f/todM-r, xylM-f/xylM-r, and ndoM-f/ndoM-r primers; and 50 °C for the 23CAT-f/23CAT-r, A24f2/A577r2, and rhlA-f/rhlB-r primers. The PCR reaction products were observed on 1.5% (*w*/*v*) agarose gel [27] by using SYBR Safe staining (Invitrogen, Carlsbad, CA, USA). The primers used in this study were purchased from Invitrogen (Carlsbad, CA, USA) and Biolegio (Malden, Netherlands).

**Degradative ability of native bacterial consortia.**

Bacterial consortia (5% *v*/*v*) were inoculated into MSM supplemented with 1–10% (*v*/*v*) diesel and incubated aerobically at 30 °C in a rotary shaker set to 200 rpm for 1–7 days. Growth of bacteria, protein profile, enzymatic profile, biosurfactant production, and diesel biodegradation were then quantified.

**Growth of bacteria.** The bacterial growth in MSM with diesel was determined by measuring the optical density at 660 nm (OD_660_) of the cultures using a SPECORD 200 UV-Vis spectrophotometer (Analytik Jena, Jena, Germany), while cell viability was assessed using the spot method [35]. Bacterial cultures (20 μL) were spotted onto LB agar [27], air-dried, and the Petri plates were incubated at 30 °C for 1–3 days. Rhodamine 6G (Rh6G) accumulation, pigment production, and lactose fermentation ability were studied using the same spot method. Bacteria that accumulated Rh6G exhibited red fluorescence on LB agar containing 10 µg mL^−1^ Rh6G. Pyocyanin-producing bacteria appeared blue-green on King A agar [36], while pyoverdine-producing bacteria fluoresced yellow-green on King B agar [36]. Lactose-fermenting bacteria produced dark purple or pink colonies on MacConkey agar.

**Protein profile.** Bacterial cells were harvested, washed twice, resuspended in 20 mM Tris-HCl buffer (pH 7.4), and sonicated. The resulting cell-free supernatant was used for total protein quantification by measuring absorbance at 280 nm (OD_280_) using a BioDrop DUO UV-Vis spectrophotometer (BioDrop Ltd., Cambridge, UK). The cell-free supernatant was mixed with gel-loading dye and denatured at 95 °C for 10 min before performing sodium dodecyl sulfate-polyacrylamide gel electrophoresis (SDS-PAGE) using a Minigel-Twin system (Biometra, Göttinger, Germany). Proteins were separated on a 12% polyacrylamide gel and stained with Coomassie Brilliant Blue R-250 [27]. The densitometry analysis of the SDS-PAGE gel was performed using the ImageJ 1.53e program.

**Enzymatic profile.** Bacterial cells were harvested, washed twice, resuspended in 20 mM Tris-HCl buffer (pH 7.4), and sonicated. The resulting cell-free supernatant (50 μL) was used for **alkane hydroxylase** activity quantification by measuring absorbance at 340 nm (OD_340_) using a SPECORD 200 UV-Vis spectrophotometer (Analytik Jena, Jena, Germany). Briefly, the reaction was initiated by adding 1% (*v*/*v*) *n*-hexadecane in 80% (*v*/*v*) DMSO and 0.1 mM NADH (as a cofactor). Alkane hydroxylase activity was expressed as 1 mmol of NADH oxidized per minute [37]. Production of extracellular **hydrolases**, such as **protease**, **lipase**, **amylase**, and **cellulase,** was studied using the spot method. Bacterial cultures (20 μL) were spotted onto wheat meal agar [38], tributyrin agar [39], starch agar [40], and carboxymethylcellulose agar [41] to assess the production of protease, lipase, amylase, and cellulase, respectively. The Petri plates were incubated at 30 °C for 1–5 days. Extracellular hydroxylase-producing bacteria were surrounded by either an emulsified or a clear halo.

**Biosurfactants.** The production of biosurfactants by bacteria was evaluated using various methods, including the hydrocarbon overlay agar, CTAB agar, methylene blue, drop-collapse, emulsification activity, biosurfactant activity, and chromatographic analysis.

Hydrocarbon overlay agar method. Bacterial cultures (20 μL) were spotted onto MSM agar plates, air-dried, and overlaid with various hydrocarbons, such as diesel, *n*-hexadecane, and *n*-heptane. The Petri plates were incubated at 30 °C for 1–5 days. Biosurfactant-producing bacteria were surrounded by an emulsified halo [42].

Cetyltrimethylammonium bromide (CTAB) agar method. Bacterial cultures (20 μL) were spotted onto CTAB methylene blue agar [43], air-dried, and the Petri plates were incubated at 30 °C for 1–5 days. A blue halo surrounded extracellular glycolipid-producing bacteria.

Methylene blue method. Bacterial cultures were mixed with chloroform (1:1, *v*/*v*) containing 0.003% (*w/v*) methylene blue, vortexed for 3 min, and biosurfactant production was determined by measuring absorbance at 625 nm (OD_625_) using a SPECORD 200 UV-Vis spectrophotometer (Analytik Jena, Jena, Germany), as described earlier [44].

Drop-collapse method. Cell-free supernatants (5 μL) were dropped onto the lid of the surface of a 96-microwell plate containing petroleum (20 μL). Biosurfactant-producing bacteria cause the petroleum drop to collapse [42].

Emulsification activity. Cell-free supernatants were mixed with diesel (1:1, *v*/*v*), vortexed for 3 min, and the emulsification index (*E*_24_) was determined after 24 h [42].

Biosurfactant activity. Cell-free supernatants were dropped onto the surface of Petri plates containing water overlaid with diesel and 0.001% (*w/v*) Sudan Black B. Biosurfactant-producing bacteria induced the formation of a clear zone on the diesel film [45].

High-performance thin-layer chromatography (HPTLC). Biosurfactants were extracted from the cell-free supernatants using a chloroform–methanol mixture [46]. HPTLC analyses were performed using a CAMAG TLC system (Muttenz, Switzerland). The samples were spotted under a nitrogen stream onto silica gel 60 TLC plates (Merck, Darmstadt, Germany). The TLC plates were developed with a chloroform–methanol–water mixture (70:25:5, *v*/*v*/*v*) and observed under UV light (366 nm) and visible light (500 nm) both before and after derivatization with iodine vapors, 0.2% (*w*/*v*) orcinol in 53% sulfuric acid, and 0.3% (*w*/*v*) ninhydrin [46].

**Biodegradation.** Diesel biodegradation was studied by observing diesel layer fragmentation and carbon dioxide (CO_2_, mg L^−1^) production [47], as well as through gravimetric and chromatographic analyses of the residual diesel extracted with chloroform from the cell-free cultures [48]. High-performance thin-layer chromatography (HPTLC) analyses were performed using a CAMAG TLC system (Muttenz, Switzerland) [35]. The samples were spotted under a nitrogen stream onto silica gel 60 TLC plates (Merck, Darmstadt, Germany). The TLC plates were developed with an *n*-hexane–ethyl acetate–water mixture (85:10:5, *v*/*v*/*v*) and observed under UV light (366 nm) and visible light (500 nm) both before and after derivatization with 3% (*w*/*v*) anisaldehyde in an acetic acid–methanol–sulfuric acid mixture (10:85:5, *v*/*v*/*v*).

## 3. Results and Discussion

**Isolation and characterization of native bacterial consortia.**

**Figure 1:**
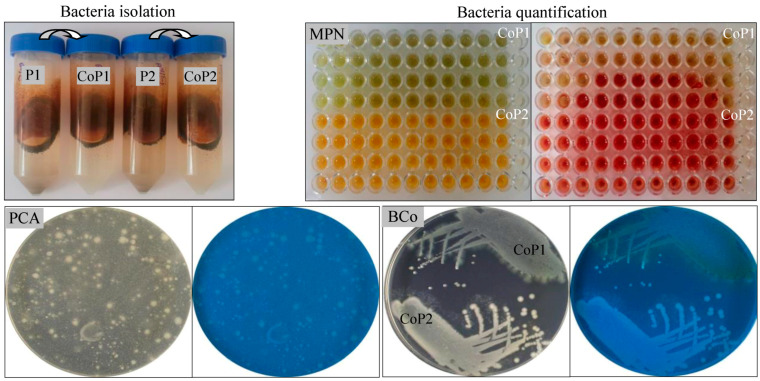
Isolation of native bacterial consortia and their quantification. Soil samples P1 and P2 used to initiate enrichment cultures in MSM-petroleum 5%; bacteria quantification in enrichment cultures by most probable number (MPN) method (microplate before and after TTC addition) and plate count agar (PCA) method; isolated bacterial consortia (BCo) CoP1 and CoP2; Petri plates observed under visible and UV light.

In countries with an extensive petroleum extraction and processing history, such as Romania, hydrocarbon contamination of soils is a significant environmental threat. Large areas of soils near extraction sites and petrochemical facilities are frequently polluted with petroleum and its derivatives. Such contamination leads to important economic losses and ecological damage. The isolation of novel bacterial consortia from polluted environments is a critical step in advancing the development and application of effective bioremediation strategies. The standard successive enrichment culture method is mainly effective for selecting bacteria capable of metabolizing hydrocarbons, as it gradually promotes the growth of organisms suitable for degrading specific pollutants [26,44,49]. Two bacterial consortia were isolated (Figure 1) from soil samples collected in Poeni, Teleorman County, Romania, a region affected by petroleum pollution. The first consortium, designated as CoP1, was isolated from a highly polluted soil sample where vegetation was severely affected. In contrast, the second consortium, CoP2, was obtained from a soil sample with lower petroleum contamination, where the vegetation showed no observable effects. It is well known that, under certain conditions, small amounts of hydrocarbons can enhance soil fertility. However, pollution with high concentrations of hydrocarbons is generally harmful to the environment, often leading to the formation of large tailings dumps. Plant life begins to suffer when the concentration of hydrocarbons in the soil exceeds 1% (*v*/*w*). Vegetation can recover if only the upper parts of the plants are affected, while when the roots are affected, the consequences of soil pollution with hydrocarbons can persist into subsequent growing seasons. As hydrocarbon concentrations rise, the carbon-to-nitrogen (C/N) ratio becomes unfavorable for sustaining life in the soil. The enrichment cultures obtained from both polluted soil samples exhibited a high bacterial number (>10^13^ cells mL^−1^), which was further supported by the plate count agar method, revealing a consistently high CFU count (>10^10^ CFU mL^−1^) for both consortia. This discrepancy between the MPN and CFU values is expected due to methodological differences. The MPN method detects metabolically active bacteria capable of growth in liquid media, including viable but non-culturable cells, while the CFU method quantifies only bacteria that form visible colonies on solid agar. Similar differences have been reported in environmental microbiology, where MPN values frequently exceed CFU counts by one or more logarithmic units. Although the CoP1 and CoP2 consortia were isolated from soil samples with different pollution levels (high and low), both demonstrated the capacity to efficiently degrade 5% (*v*/*v*) petroleum, achieving a biodegradation rate over 65–70%. Therefore, these consortia are probably composed of bacteria adapted to use hydrocarbons as their sole carbon and energy source, making them excellent candidates for further study. To confirm the presence of bacteria in the CoP1 and CoP2 consortia, the extracted genomic DNA was used as a template for PCR amplification of the 16S rRNA gene. The expected 1465 bp fragment of the bacterial 16S rRNA gene was successfully detected in the DNA extracted from both consortia (Table 1). These findings align with previous hypotheses, indicating that microbial communities in petroleum-contaminated soils are predominantly composed of bacteria adapted to survive in the presence of toxic hydrocarbons and utilize them as metabolic substrates for growth. The functional and species diversity of such bacterial communities is typically influenced by the type and concentration of pollutants [15].

Different phenotypic traits were observed between the two isolated consortia (Table 1). The CoP1 consortium exhibited growth within a temperature range of 20–40 °C, whereas the CoP2 consortium grew within a narrower range of 25–40 °C. Both consortia demonstrated optimal growth at 30 °C. On LB agar, the CoP1 consortium produced yellow-green pigmented colonies, while the CoP2 consortium formed cream-colored colonies. Through optical microscopy observation, we established that the CoP1 consortium contains motile, rod-shaped Gram-negative, and nonmotile, cocci-shaped Gram-positive bacteria. In contrast, the CoP2 consortium contains only motile, rod-shaped Gram-negative bacteria. These observations further support phenotypic diversity within the two isolated consortia. Both consortia demonstrated facultative anaerobic growth under aerobic conditions and accumulated rhodamine 6G, an indicator of active membrane transport mechanisms potentially linked to hydrocarbon degradation. On King A and King B agar, the CoP1 consortium produced both pyocyanin and pyoverdine (siderophores known to enhance survival in polluted sites), whereas CoP2 exhibited only weak fluorescence, suggesting differences in iron acquisition and oxidative stress responses. The growth of the CoP1 consortium on TTC agar provides evidence of the presence of *Pseudomonas aeruginosa* within it. On MacConkey agar, the CoP1 consortium formed dark purple colonies, while the CoP2 consortium produced pink, mucoid colonies, indicating the presence of lactose-fermenting bacteria in both consortia. Additionally, both consortia synthesized extracellular hydrolases, such as protease, lipase, amylase, and cellulase, which play a key role in hydrocarbon biodegradation [50]. They also exhibited robust viability when LB agar was overlaid with 100% diesel, gasoline, kerosene, *n*-hexadecane, *n*-pentadecane, *n*-decane, *n*-heptane, *n*-hexane, xylene isomers, ethylbenzene, and toluene. These findings indicate that both consortia harbor bacteria capable of tolerating complex hydrocarbon mixtures and individual pure hydrocarbons. Our results are consistent with previous studies suggesting that hydrocarbon-degrading bacterial consortia undergo distinct phenotypic adaptations influenced by environmental factors and interspecies interactions. Consortia isolated from hydrocarbon-contaminated environments demonstrate high adaptability, comprising bacteria capable of tolerating complex hydrocarbon mixtures and individual pure hydrocarbons. Furthermore, Gram-negative bacteria, particularly those of the *Pseudomonas* genus, often dominate these consortia due to their remarkable metabolic versatility [6,11,14,16].

The genes encoding enzymes of the catabolic pathway for hydrocarbon degradation are critical for the ability of bacteria to bioremediate soils polluted with petroleum and its derivatives and have been identified in both Gram-positive and Gram-negative bacteria [6]. The ability of the two isolated consortia to degrade hydrocarbons was confirmed by the presence of several well-known catabolic genes in their genome, such as *alkB* (expected size fragment 870 bp) and *alkM* (870 bp), which encode alkane hydroxylases that initiate alkane degradation, as well as *todM* (560 bp), *xylM* (834 bp), *ndoM* (642 bp), and *C23DO* (238 bp), which encode various dioxygenases or monooxygenase involved in the degradation of aromatic hydrocarbons (Table 1). In the DNA extracted from the CoP1 consortium, four catabolic genes (*alkB*, *alkM*, *todM*, *ndoM*) were detected, while only two genes (*ndoM*, *C23DO*) were found in the genome of the CoP2 consortium. The resistance-nodulation-cell division (RND) transporter gene (*HAE1*, 550 bp) was detected in the DNA extracted from both consortia. In contrast, the rhamnosyltransferase 1 genes (*rhlAB*, 216 bp), involved in rhamnolipid biosynthesis, were detected in the DNA extracted from CoP1, while in the CoP2 consortium, these genes were not found. In nature, thousands of bacterial genera and species can utilize hydrocarbons as carbon and energy sources. The bioremediation of hydrocarbon-polluted soils requires the combined action of bacteria that possess multiple catabolic genes, which encode degradative enzymes such as oxygenases (monooxygenases and dioxygenases), dehydrogenases, hydroxylases, and peroxidases, all important for the complete degradation of *n*-alkanes and aromatic hydrocarbons [6,11].

**Degradative ability of native bacterial consortia.**

**Figure 2:**
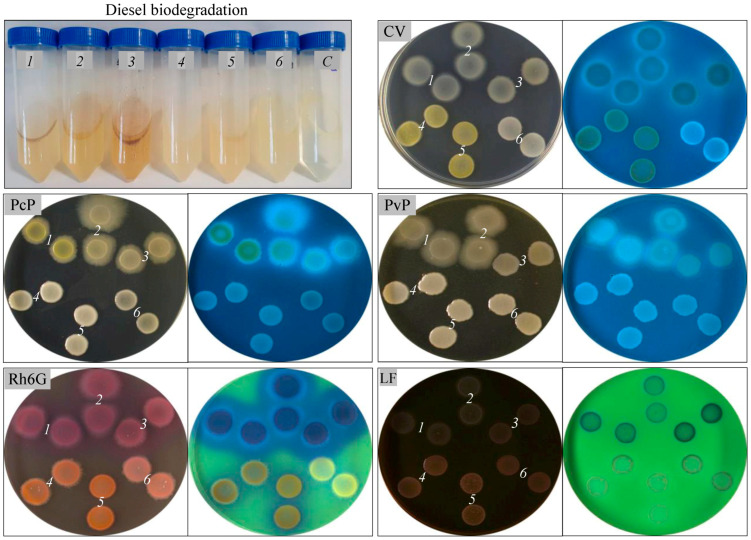
Biodegradation of diesel by native bacterial consortia. CoP1 cultured in MSM-diesel 1% (*1*), 5% (*2*), 10% (*3*); CoP2 cultured in MSM-diesel 1% (*4*), 5% (*5*), 10% (*6*); control (C, uninoculated medium). Cell viability (CV); pyocyanin (PcP) and pyoverdine (PvP) production; Rh6G accumulation; lactose fermentation (LF); Petri plates observed under visible and UV light.

Both consortia demonstrated robust growth on the MSM agar overlaid with 100% diesel for 24 h. These consortia exhibited good tolerance to 1–10% diesel when this complex hydrocarbon mixture was used as a sole carbon and energy source in liquid MSM (Table 2, Figure 2). The CoP1 consortium showed higher growth (OD_660_ 0.84–1.29) in the presence of diesel, compared with that of CoP2 (0.67–1.15). The bacterial growth rate varied from one consortium to another (OD_660_ 0.67–1.29) depending on the diesel concentration. A higher bacterial growth rate (OD_660_ 0.74–1.29) was observed when diesel was used at lower concentrations (1%, 5%), compared with their growth (0.84, 0.67) when this complex hydrocarbon mixture was used at a higher concentration (10%). The bacterial growth rate of both consortia decreases with the increase in the diesel concentration in the MSM. Although differences in the bacterial growth rates were observed (with the OD_660_ increasing 10 to 16 times compared to the initial values), all of the cultures grown for 7 days in MSM with 1–10% diesel demonstrated good viability, showing good growth when spotted onto LB agar (Table 2, Figure 2). Abundant growth was also observed on King A and King B agar for the cultures grown in MSM with 1–10% diesel. The CoP1 consortium produced pyocyanin and pyoverdine on King A and King B agar, while CoP2 revealed only weak fluorescence. When bacterial cultures were spotted on LB agar containing Rh6G, they exhibited red fluorescence under UV light, indicating the accumulation of rhodamine 6G. This accumulation by bacteria in the CoP1 and CoP2 consortia suggests the presence of P-glycoprotein in their membranes. The presence of this ABC transporter in both consortia is expected, as it plays a key role in exporting hydrophobic compounds (e.g., hydrocarbons) from cells, thereby preventing their intracellular accumulation at high concentrations [12]. The CoP1 consortium, cultured in MSM containing 1–10% diesel, produced dark purple colonies on MacConkey agar, while the CoP2 consortium formed pink mucoid colonies. These colony characteristics confirm the presence of lactose-fermenting bacteria with hydrocarbon-degrading capabilities in both consortia. Pollution with petroleum and its derivatives in soil reduces the species richness, evenness, and phylogenetic diversity, leading to a bacterial community structure dominated by a few hydrocarbon-degrading bacterial species. Up to now, over 80 genera of bacteria, such as *Achromobacter*, *Acinetobacter*, *Arthrobacter*, *Burkholderia*, *Enterococcus*, *Enterobacter*, *Pseudomonas*, *Serratia*, and *Rhodococcus,* capable of degrading hydrocarbons, have been isolated from different environments [11]. Among these, *Pseudomonas* sp. is well recognized as an ideal candidate for hydrocarbon biodegradation due to its remarkable ability to degrade and utilize various toxic hydrocarbons, including those from diesel [16,22].

**Figure 3:**
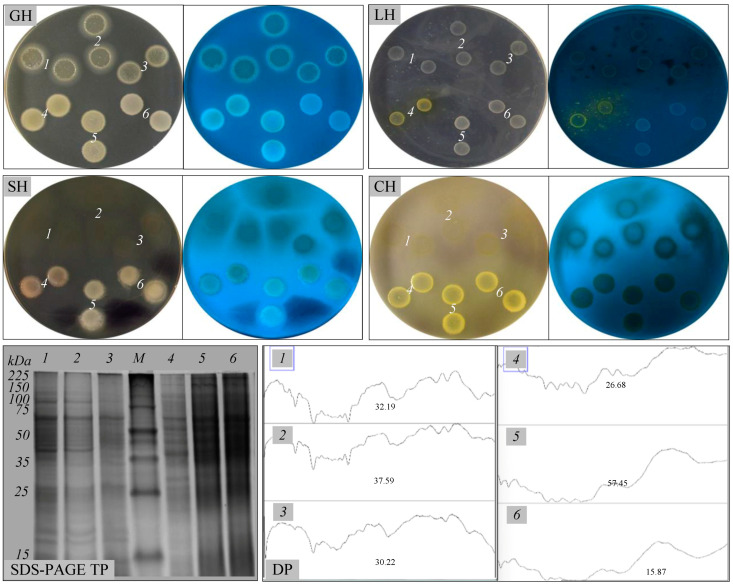
Enzymatic and protein profile of native bacterial consortia. CoP1 cultured in MSM-diesel 1% (*1*), 5% (*2*), 10% (*3*); CoP2 cultured in MSM-diesel 1% (*4*), 5% (*5*), 10% (*6*). Protease (gelatin hydrolysis, GH); lipase (lipid hydrolysis, LH); amylase (starch hydrolysis, SH); cellulase (cellulose hydrolysis, CH); Petri plates observed under visible and UV light. SDS-PAGE of total-cell protein (TP), broad-range protein molecular weight marker, Promega (M); densitometry plots (DP) for the SDS-PAGE gel.

As a result of the bacterial growth, the total protein concentration increased 6- to 8-fold (0.33–0.39 mg mL^−1^) after 7 days of incubation in MSM containing 1–10% diesel, compared to the initial concentration. The changes induced by the presence of diesel on the whole-cell proteins were further studied by electrophoresis (Figure 3). We observed changes in the cellular proteome when the CoP1 and CoP2 consortia were grown in the presence of 1–10% diesel as the sole carbon and energy source. Proteins with molecular weights between 15 and 225 kDa were detected in the whole-cell protein extracts of these consortia. The growth of the CoP1 consortium in the presence of diesel resulted in the production of proteins with molecular weights ranging from 15 to 100 kDa. In contrast, the CoP2 consortium exhibited protein production at molecular weights ranging from 25 to 225 kDa. Both SDS-PAGE studies and densitometric analysis (DP, plot areas 30.22-37.59 for CoP1 and 15.87-57.45 for CoP2) of gel images indicated alterations in the protein expression patterns of the two bacterial consortia grown in the presence of 1–10% diesel. Hydrocarbons are well known to have harmful effects on bacteria and their proteins, directly or indirectly linked to morphological changes and cellular stress responses. The induction and repression of several proteins, such as membrane-embedded proteins, chemotaxis proteins, and chaperones, in response to stress conditions, like exposure to pure or mixtures of hydrocarbons, have already been reported [12,16,51].

Alkane hydroxylase production was also analyzed in the two bacterial consortia cultured in MSM containing 1–10% diesel (Table 2). Both consortia revealed good alkane hydroxylase activity, and the enzyme activity was different for the two consortia depending on the diesel concentration used (ranging from 34.49 to 73.87 U mL^−1^). Higher alkane hydroxylase activity (73.87, 71.07 U mL^−1^) was obtained for the CoP1 consortium when grown in the presence of lower diesel concentrations (1%, 5%), compared to the activity measured at a higher diesel concentration (10%), which was 55.11 U mL^−1^. A similar trend was observed for the CoP2 consortium, where higher enzyme activities (70.87, 61.37 U mL^−1^) were recorded at 1% and 5% diesel, and lower activity (34.49 U mL^−1^) was noted at the higher diesel concentration of 10%. These results were not unexpected, since the hydroxylation activity of this enzyme is regulated by the presence of a substrate (*n*-alkanes) and co-factor (NADH) [52]. Aliphatic hydrocarbons, like *n*-alkanes, which are major components of diesel, are aerobically broken down by several groups of enzymes, such as alkane hydroxylase, alcohol dehydrogenase, aldehyde dehydrogenase, esterase, and lipase. From these groups, alkane hydroxylase is known to be the first enzyme that initiates the aerobic degradation of *n*-alkanes [53] to alcohols, which are further oxidized to fatty acids, and then catabolized via the β-oxidation pathway [54]. The small- and medium-chain *n*-alkanes are oxidized frequently by methane monooxygenase and non-heme alkane monooxygenase. In contrast, long-chain *n*-alkanes (over C_20_) are oxidized by several enzymes, such as alkane hydroxylase, cytochrome P450 monooxygenases (heme-thiolate proteins), flavin monooxygenase, and long-chain alkane monooxygenase LadA [37]. As expected, both consortia grown in MSM containing 1–10% diesel exhibited good growth when spotted onto wheat meal agar, starch agar, carboxymethylcellulose agar, or tributyrin agar. Like other bacteria isolated from hydrocarbon-polluted sites, the CoP1 and CoP2 consortia produced extracellular hydrolases, such as protease, amylase, cellulase, and lipase (Table 2, Figure 3). Enzyme production was not affected by increasing the diesel concentration in the MSM from 1% to 10%. Earlier studies have reported that bacteria from hydrocarbon-polluted sites frequently produce extracellular hydrolases, which facilitate the degradation of petroleum and its derivatives. Acting as biocatalysts, these enzymes mediate the hydrolytic cleavage of esters, amides, and other chemical bonds within hydrocarbon structures, enhancing bacterial degradation processes [50].

**Figure 4:**
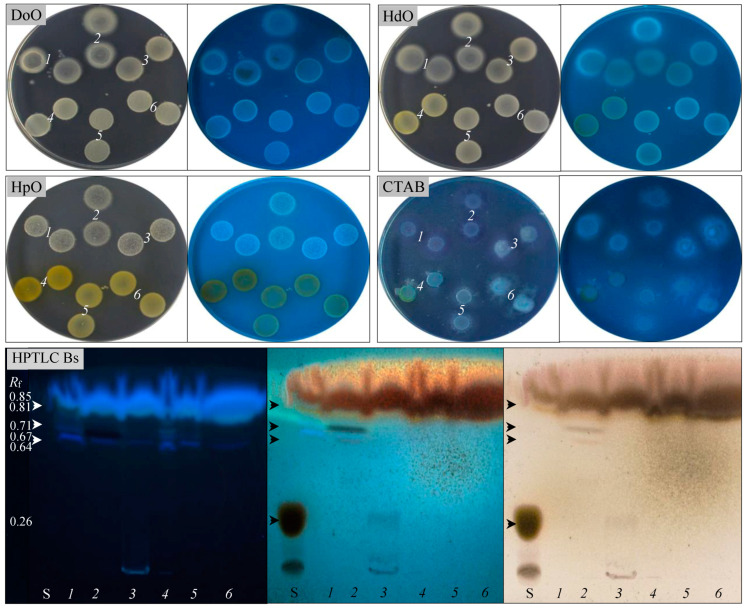
Biosurfactant production by native bacterial consortia. CoP1 cultured in MSM-diesel 1% (*1*), 5% (*2*), 10% (*3*); CoP2 cultured in MSM-diesel 1% (*4*), 5% (*5*), 10% (*6*). Diesel overlay (DoO), *n*-hexadecane overlay (HdO) and *n*-heptane overlay (HpO); CTAB agar; Petri plates observed under visible and UV light. HPTLC analysis of biosurfactants (Bs), showing the retardation factor (*R*_f_) of chromatographic peaks (arrows) and the sugar standard L-rhamnose (S); TLC plates observed under UV (left, middle) and visible (right) light.

Biosurfactants are surface-active molecules synthesized by various bacterial species, either associated with the cell surface or secreted extracellularly. The ability of the CoP1 and CoP2 consortia to synthesize secondary metabolites, such as biosurfactants, was also studied during cultivation in MSM containing 1–10% diesel (Table 2, Figure 4). The hydrocarbon overlay agar method confirmed biosurfactant production by CoP1 and CoP2 consortia. Similarly, when the CTAB blue agar plate method was used as a screening method, CoP1 and CoP2 showed positive results, indicating that the bacteria in these consortia produce extracellular glycolipids or other anionic surfactants. These observations were further supported by the methylene blue method (OD_625_ 1.23–2.96), the drop-collapse method, emulsification activity (*E*_24_ 60–85%), and biosurfactant activity (clear zone diameter on the diesel film 65–87 mm). The results are consistent with previous studies on bacterial consortia isolated from hydrocarbon-polluted environments, emphasizing their ability to produce biosurfactants while facilitating hydrocarbon degradation [14,18,21]. Similarly, Cameotra and Singh [55] reported the isolation of a bacterial consortium from oily sludge-contaminated soil, consisting of two *Pseudomonas aeruginosa* isolates and one *Rhodococcus erythropolis* isolate, which demonstrated the ability to degrade 90% of hydrocarbons within six weeks.

The crude biosurfactants produced by the CoP1 and CoP2 consortia were finally analyzed by chromatographic analyses (Figure 4). When the chromatogram was observed under UV light (before derivatization), two and three distinct fractions with different migration properties (*R*_f_ 0.64–0.67, 0.70–0.71, and 0.81–0.85) were obtained for the CoP1 and CoP2 consortia. One to three fractions with varying migration properties (*R*_f_ 0.26, 0.67, 0.71, and 0.81–0.85) were also obtained when the chromatogram was observed under UV or visible light, following plate derivatization with iodine vapors or orcinol. Biosurfactants extracted from CoP1 and CoP2 grown in the presence of diesel showed positive reactions with iodine vapors, which indicate the presence of lipids, as well as positive reactions with orcinol, which indicate the presence of sugars in their molecules. Negative reactions with ninhydrin indicate the absence of proteins with amino groups in the produced biosurfactants. Thus, these biosurfactants are glycolipids, and they were extracellularly secreted by the bacteria that exist in the CoP1 and CoP2 consortia. Our results align with previously reported data, indicating that various bacterial consortia isolated from hydrocarbon-polluted sites are typically excellent biosurfactant producers, capable of enhancing hydrocarbon solubility and promoting their biodegradation [2,14,18,19,21,52,55].

**Figure 5:**
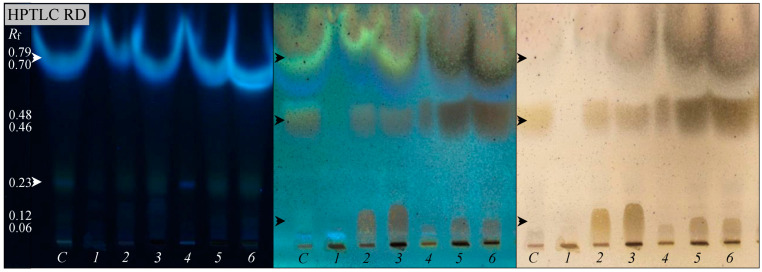
Biodegradation of diesel by native bacterial consortia. CoP1 cultured in MSM-diesel 1% (*1*), 5% (*2*), 10% (*3*); CoP2 cultured in MSM-diesel 1% (*4*), 5% (*5*), 10% (*6*); control (C, uninoculated medium). HPTLC analysis of residual diesel (RD), showing the retardation factor (*R*_f_) of chromatographic peaks (arrows); TLC plates observed under UV (left, middle) and visible (right) light.

Diesel biodegradation by bacterial consortia isolated from hydrocarbon-polluted sites is widely recognized as a promising and environmentally friendly approach to managing diesel pollution. The diesel degradation capacity of the CoP1 and CoP2 consortia was evaluated when cultured in MSM containing 1–10% diesel (Table 2). The production of CO_2_ presented high values (1300–1840 mg L^−1^) for both consortia and the released CO_2_ quantity increased with the increase in the diesel concentration in MSM. No CO_2_ release was observed for the control (uninoculated medium with diesel). These observations were confirmed by diesel layer fragmentation and gravimetric analyses of the residual diesel. The CoP1 and CoP2 consortia degraded 1–10% diesel in 7 days. The biodegradation rate differs from one consortium to another (50.81–84.32%), and even for the same consortium depending on the diesel concentration. Like in the growth assay, the biodegradation rate of these consortia decreases with the increase in the diesel concentration in the medium. A higher biodegradation rate (60.74–84.32%) was observed when diesel was used at lower concentrations (1%, 5%), compared with their biodegradation (59.74%, 50.81%) when diesel was used at a higher concentration (10%). These observations were confirmed by the chromatographic analyses of the residual diesel (Figure 5). When the chromatogram was observed under UV light (before plate derivatization), two spots with different migration properties (with an *R*_f_ of 0.23 to 0.70–0.79) were obtained for the control and the CoP1 and CoP2 consortia. Previously, Ko et al. [56] reported that the differences observed in spot shapes and migration properties reflect the varying polarities of compounds found in complex hydrocarbon mixtures, such as diesel. When the chromatogram was observed under UV or visible light after plate derivatization with anisaldehyde, up to three distinct spots (*R*_f_ 0.06–0.12 to 0.70–0.79) were detected, each exhibiting unique shapes and migration properties. When the CoP1 consortium was grown in MSM with 1% diesel, only one spot (*R*_f_ 0.76) was observed, whereas when these bacteria were grown in 5% and 10% diesel, three spots (*R*_f_ 0.10–0.12, 0.46, and 0.74–0.79) were detected. Similarly, three spots (*R*_f_ 0.06–0.08, 0.48, and 0.70–0.79) were observed when the CoP2 consortium was grown in low and high diesel concentrations. Gravimetric and chromatographic analyses of the residual diesel confirm that the CoP1 and CoP2 consortia can degrade hydrocarbons found in diesel. Additionally, we observed a direct relationship between the growth of the two isolated bacterial consortia in the presence of 1–10% diesel and the biodegradation of diesel (Table 2). The CoP1 consortium exhibited higher growth (OD_660_ 0.84–1.29) and diesel biodegradation efficiency (59.74–84.32%) compared to those of CoP2 (OD_660_ 0.67–1.15, 50.81–79.21%), suggesting differences in metabolic capacity and bacterial composition. Similarly, Moliterni et al. [15] reported the isolation of three bacterial consortia from polluted soils and observed a direct relationship between bacterial growth and diesel depletion for all of them. The maximum diesel consumption by consortia XA, XB, and XC occurred during the exponential growth phase, where the biomass concentration increased as the diesel levels rose from 0.5% to 3% [15].

The majority of the hydrocarbon constituents in diesel are biodegraded by bacteria that normally occur in soil. However, the bioremediation of diesel-polluted soil is often limited by the low biodiversity of specialized native bacteria, which lack the complementary substrate specificity required for the complete degradation of such complex hydrocarbon mixtures [5,8,13]. According to the literature, bacteria capable of producing biosurfactants and demonstrating enhanced hydrocarbon-degrading capacities are widely recognized as key agents for achieving rapid and efficient hydrocarbon degradation in contaminated environments, making them indispensable for the development of effective bioremediation strategies [2,4,18,19,57].

## 4. Conclusions

Petroleum spills resulting from exploration, transport, and processing have become a significant environmental issue in many countries, including Romania. Using the standard successive enrichment culture method, two native bacterial consortia, CoP1 and CoP2, were successfully isolated from hydrocarbon-polluted soils. These consortia exhibited a good tolerance to diesel, as well as to other complex hydrocarbon mixtures like gasoline and kerosene, and pure hydrocarbons such as *n*-hexadecane, *n*-pentadecane, *n*-decane, *n*-heptane, *n*-hexane, xylene isomers, ethylbenzene, and toluene. The CoP1 consortium revealed higher growth (OD_660_ 0.84–1.29) and diesel biodegradation efficiency (59.74–84.32%) compared to those of CoP2 (OD_660_ 0.67–1.15, 50.81–79.21%), suggesting differences in metabolic capacity and bacterial composition. The production of glycolipid-type biosurfactants and extracellular enzymes (protease, amylase, cellulase, and lipase) by both consortia highlights their potential to enhance hydrocarbon solubilization and degradation, a crucial factor in bioremediation. The significant alkane hydroxylase activity (34.49–73.87 U mL^−1^) and the presence of key catabolic genes (*alkB*, *alkM* for alkane degradation, and *todM*, *ndoM*, *C23DO* for aromatic hydrocarbon degradation) confirm the metabolic versatility of these consortia in hydrocarbon breakdown. The detection of the *HAE1* gene in both consortia and the presence of *rhlAB* genes only in CoP1 suggest functional differences in biosurfactant synthesis, which may explain its enhanced biodegradation performance. These findings indicate that the CoP1 and CoP2 consortia are well adapted for the efficient biodegradation of hydrocarbons. Their ability to degrade diesel and produce biosurfactants underscores their strong potential for application in the bioremediation of soils contaminated with petroleum and its derivatives. Further studies should focus on optimizing environmental conditions to maximize their degradation efficiency in real-world scenarios.

## Figures and Tables

**Table 1:** Phenotypic and molecular characterization of native bacterial consortia.

Characteristics	Consortium	
	CoP1	CoP2
Phenotypic traits		
Temperature growth (°C)	30 (20–40)	30 (25–40)
Pigmentation	yellow-green	cream
Gram	−, +	−
Cell shape	rods, cocci	rods
Motility	+, −	+
Facultative anaerobic growth	+	+
Rh6G accumulation	+	+
Pyocyanin production	+	−
Pyoverdine production	+	+
Tolerance to TTC	+	−
Lactose fermentation	+	+
Protease (gelatin hydrolysis)	+	+
Lipase (lipid hydrolysis)	+	+
Amylase (starch hydrolysis)	+	+
Cellulase (cellulose hydrolysis)	+	+
Hydrocarbon tolerance traits		
Complex mixtures (diesel, gasoline, kerosene)	+	+
*n*-Alkanes (*n*-hexadecane, *n*-pentadecane, *n*-decane, *n*-heptane, *n*-hexane)	+	+
Aromatics (xylene isomers, ethylbenzene, toluene)	+	+
Molecular traits		
Bacterial 16S rRNA gene (1465 bp)	+	+
Catabolic genes		
Alkane hydroxylase *alkB* (870 bp)	+	−
Alkane hydroxylase *alkM* (870 bp)	+	−
Toluene dioxygenase *todM* (560 bp)	+	−
Toluene/xylene monooxygenase *xylM* (834 bp)	−	−
Naphthalene dioxygenase *ndoM* (642 bp)	+	+
Catechol 2,3-dioxygenase *C23DO* (238 bp)	−	+
RND transporter *HAE1* gene (550 bp)	+	+
Rhamnosyltransferase 1 *rhlAB* genes (216 bp)	+	−

Phenotypic traits, including physiological and biochemical traits; hydrocarbon tolerance traits, such as bacterial growth on LB agar overlaid with complex hydrocarbon mixtures, *n*-alkanes, or aromatics; molecular traits, such as PCR amplification of the bacterial 16S rRNA gene, catabolic genes, RND transporter gene, and rhamnosyltransferase 1 genes; positive reaction (+); negative reaction (−).

**Table 2:** Biodegradation of diesel by native bacterial consortia.

**Biodegradation assay**	**Consortium**					
	**CoP1** cultured in MSM-diesel			**CoP2** cultured in MSM-diesel		
	1%	5%	10%	1%	5%	10%
Bacterial growth quantification						
Growth (OD_660_)	1.29 ± 0.05	1.06 ± 0.08	0.84 ± 0.04	1.15 ± 0.04	0.74 ± 0.06	0.67 ± 0.07
Cells viability	+	+	+	+	+	+
Rh6G accumulation	+	+	+	+	+	+
Pyocyanin production	+	+	+	−	−	−
Pyoverdine production	+	+	+	+	+	+
Lactose fermentation	+	+	+	+	+	+
Enzymes						
Alkane hydroxylase (U mL^−1^)	73.87 ± 0.08	71.07 ± 0.09	55.11 ± 0.12	70.87 ± 0.10	61.37 ± 0.13	34.49 ± 0.11
Protease (gelatin hydrolysis)	+	+	+	+	+	+
Lipase (lipid hydrolysis)	+	+	+	+	+	+
Amylase (starch hydrolysis)	+	+	+	+	+	+
Cellulase (cellulose hydrolysis)	+	+	+	+	+	+
Biosurfactants						
Hydrocarbon overlay agar	+	+	+	+	+	+
CTAB agar	+	+	+	+	+	+
Methylene blue (OD_625_)	2.96 ± 0.07	2.84 ± 0.06	1.97 ± 0.09	1.57 ± 0.05	1.41 ± 0.09	1.23 ± 0.07
Drop-collapse	+	+	+	+	+	+
Emulsification activity (%)	85	79	66	71	64	60
Biosurfactants activity (mm)	87	80	75	74	68	65
Biodegradation						
Diesel layer fragmentation	+	+	+	+	+	+
CO_2_ production (mg L^−1^)	1520 ± 0.12	1630 ± 0.15	1840 ± 0.10	1300 ± 0.14	1400 ± 0.13	1670 ± 0.09
Diesel biodegradation (%)	84.32 ± 0.13	72.57 ± 0.10	59.74 ± 0.10	79.21 ± 0.12	60.74 ± 0.10	50.81 ± 0.15

Growth (OD_660_), expressed as the maximum optical density at the beginning of the stationary phase ± standard deviation (SD); alkane hydroxylase (U mL^−1^) activity ± SD; methylene blue assay for biosurfactant extracts (OD_625_) ± SD; CO_2_ production (mg L^−1^) ± SD; diesel biodegradation (%) ± SD; positive reaction (+); negative reaction (−).

## Data Availability

The data presented in this study are available on reasonable request.

## Statistical Analysis

All laboratory-based experiments in this study were performed in duplicates. Statistical analyses, such as the mean (±) standard deviation (SD) of each duplicate, were calculated using Microsoft Excel 2021 on a Windows 10 operating system.
